# Educational Case: Mechanism and locations of intracranial hypertensive hemorrhage leading to stroke

**DOI:** 10.1016/j.acpath.2025.100165

**Published:** 2025-02-19

**Authors:** Joshua Richmond, Jake Quarles, Sanjay Talati, Jyotsna Pandey

**Affiliations:** a Central Michigan University College of Medicine, 1280 East Campus Drive, Mount Pleasant, MI, USA; b Advanced Radiology Services, P.C., Mount Pleasant, MI, USA

**Keywords:** Pathology competencies, Organ system pathology, Nervous system-central nervous system ischemia of the brain, Hypertensive hemorrhage, Intracerebral hemorrhage, Hyaline arteriosclerosis, Charcot–Bouchard microaneurysm

## Primary objective

Objective NSC7.4: Hypertensive Hemorrhage. Describe the mechanism of hypertensive hemorrhage and name common locations in which this occurs.

Competency 2: Organ System Pathology; Topic NSC: Nervous System-Central Nervous System; Learning Goal 7: Ischemia of the Brain.

The following fictional case is intended as a learning tool within the Pathology Competencies for Medical Education (PCME), a set of national standards for teaching pathology. These are divided into three basic competencies: Disease Mechanisms and Processes, Organ SystemPathology, and Diagnostic Medicine and Therapeutic Pathology. For additional information, and a full list of learning objectives for all three competencies, see https://www.journals.elsevier.com/academic-pathology/pathology-competencies-for-medical-education-pcme.^1^

## Patient presentation

A 61-year-old man is brought to the emergency department by his wife after he suddenly began vomiting and appeared confused. The patient has slurring of his words and also has nausea, headache, and left sided weakness. The patient is noted to be right-handed. The past medical history is significant for poorly controlled hypertension. His medications include losartan 50 mg (an angiotensin receptor blocker) and 75 mg aspirin taken orally once a day. There is no relevant or significant family history. There is no history of alcohol, tobacco, or recreational drug use.

## Diagnostic findings, Part 1

Vital signs are significant for a blood pressure of 190/110 mmHg, a heart rate of 40 beats per minute, and a respiration rate of 20 per minute. No other significant findings are noted on respiratory and cardiac examination.

Neurologic motor examination reveals left-sided hemiplegia with hyperactive deep tendon reflexes, as well as a rightward deviation of the eyes. Lower left facial weakness is also noted. Babinski sign is positive. Sensation is intact for light touch and pinprick bilaterally.

## Questions/discussion points, Part 1

### What is the differential diagnosis based on the history and physical exam?

The patient has a sudden onset of clinical symptoms and is showing signs of acute focal neurologic impairment. The acute onset of symptoms is suggestive of a stroke. The most common cause of stroke in this age group is cerebral infarction (80–85%), most of the remaining will be caused by an intracerebral hemorrhage.^2^ Some of the uncommon causes that may be included in differential diagnosis as they may mimic the stroke are primary or metastatic cerebral tumors, subdural or extradural hematoma, demyelinating diseases, and cerebral abscess. Additionally certain disorders may “functionally” mimic a stroke and these include postepileptic paresis, hypoglycemia, migraine aura, focal seizures, vestibular disorders like Meniere disease, conversion disorder, and encephalitis.^2^

Cerebral infarction develops insidiously over time, and this may be an important clue to clinically determining the cause of the stroke. With a hemorrhagic stroke, there is explosive hemorrhage into the brain tissue that leads to immediate loss of function followed by sudden and acute precipitation of signs and symptoms. This is not an absolute distinction, as large artery thromboembolic infarction may have an acute presentation. Additionally, once the developing infarction leads to complete obstruction to blood flow, symptoms develop rapidly, and at this stage of presentation it may be difficult to distinguish between hemorrhagic stroke and infarction. In patients presenting with severely elevated blood pressure with a history of uncontrolled hypertension, suspicion of hemorrhagic stroke should be high. Thus, focal neurologic impairment in combination with severely elevated blood pressure on a background of chronic uncontrolled hypertension points toward hypertensive hemorrhagic stroke.^3^^,^^4^ Vomiting is also more commonly seen in hemorrhagic stroke compared to stroke due to ischemic causes.^3^ Although hypertension is the most common cause associated with hemorrhagic stroke, a number of less common pathologies that may lead to intracerebral hemorrhage must be considered. They include amyloid angiopathy (CAA), impaired blood coagulation due to anticoagulant therapy or coagulation dyscrasias, or thrombolytic therapy, arteriovenous malformations, hemangiomas, and substance misuse involving amphetamines and cocaine, etc.^2^^,^^5^^,^^6^ A summary of different types of strokes along with associated pathologies and causes is provided in Table 1 along with a differentiation between the “lobar hemorrhages” and hemorrhages in the basal ganglion, thalamus, and pons causing hemorrhagic stroke.

**Table 1:** Categories, associated pathology and causes of various types of strokes.

Type of stroke	Ischemic stroke		Hemorrhagic stroke				
Lesion	**Pale Infarct**	**Hemorrhagic Infarct**	**Deep Hemorrhage:** Basal Ganglia, Thalamus, Pons		**Lobar Hemorrhage:** Cerebral and Cerebellar Hemispheres		
Pathology	**Arterial Thromboembolism**	**Venous Thromboembolism**	**Hypertensive injury**	**Toxic-metabolic**	**Neoplastic**	**Developmental**	**Toxic-metabolic**
Causes	**Cardioembolic:** Atrial fibrillation Myocardial infarction prosthetic valve rheumatic heart disease Ischemic cardiomyopathy	**Hypercoagulable disorders:** Protein S deficiency Protein C deficiency Factor V leiden homocysteinemia SLE and antiphopholipid syndrome Oral contraceptive use **Venous sinus thrombosis**	**Hyaline Arteriosclerosis Charcot-Bouchard Aneurysm**	**Exogenous:** Cocaine	**Primary:** Glioblastoma **MMetastatic:** Carcinoma melanoma germ Cell Neoplasm	**Vascular Malformations:** Arteriovenous malformation cavernous hemangioma	**Endogenous:** Amyloid Angiopathy
	**Artery to Artery:** Atherosclerosis dissection of internal carotid or vertebral artery e.g. due to Marfan syndrome, or Other vascular pathologies						

Bold has been done to give better readability and draw attention to grouping of the disorders.

### What investigations should be ordered at this time and why?

It is most important to differentiate hemorrhagic from ischemic stroke because initial treatment for ischemic stroke includes thrombolytic therapy and anticoagulation, which are contraindicated in hemorrhagic stroke. Ccomputed tomography (CT) or magnetic resonance imaging (MRI) are important diagnostic tools in differentiating hemorrhagic stroke from ischemic stroke and determining the course of treatment.^7^ Head CT may be preferred as it requires less time and is also suitable for patients with metal implants, unlike MRI, which requires more time to complete and is contraindicated in patients with metal implants. It is important to reiterate here that CT scans of hemorrhage within neoplasms can be misinterpreted as hemorrhages, with MRI (when performed) clarifying the presence of a mass lesion along with hemorrhage in most cases.

The radiologic picture of hemorrhagic stroke shows a hyperdense area in the location of the acute bleed on head CT. Blood from an acute hemorrhage later becomes isodense and may be seen as a ring-enhancing lesion over the ensuing days and weeks. In the case of hemorrhagic stroke, urgent imaging is important as it helps in determining the extent of spread of the blood into the ventricles, predicting and tracking the progression of hematoma enlargement, exploring for the presence of surrounding edema, and evaluation for the presence of brain herniation.^8^ Subsequent imaging may be necessary in the event of worsening clinical signs to evaluate for hematoma expansion or rebleed.^8^^,^^9^

Ischemic thrombotic strokes, on the other hand, appear initially as a hyperdense segment of a vessel and progress with a loss of gray-white matter differentiation, along with hypoattenuation of the deep nuclei. Cortical hypodensity may also be appreciated.^9^ An important feature that can sometimes make the differentiation between ischemic and hemorrhagic stroke difficult is that hemorrhage can occur in the area of ischemic necrosis, and if there is significant hemorrhage, it may be difficult to differentiate from primary hemorrhagic pathology both clinically and radiologically.^2^

Other investigations useful in the evaluation of acute hemorrhagic stroke include complete blood count, serum electrolytes, renal function screen with BUN, creatinine, and urinalysis. Serum glucose should be done to assess for diabetes mellitus. Hemostasis is assessed by prothrombin time (PT) along with international normalized ratio (INR) and activated partial thromboplastin time (APTT) to determine the patient's coagulation status. A pregnancy test in female patients of childbearing age is warranted to exclude pregnancy-associated causes of stroke. Pregnancy-associated stroke, although usually seen in late pregnancy, can occur at any stage. Ischemic strokes are more common and due to pregnancy-associated thromboembolism. Hemorrhagic strokes can also occur in pregnancy due to congenital or acquired vessel wall abnormalities.^10^ Additionally, cardiac-specific troponin to evaluate for concomitant cardiac ischemia may be done. A toxicology screen to detect sympathomimetic drugs may be done as there is an associated risk between hemorrhagic stroke and phenylpropanolamine or substances of abuse such as cocaine.^11^

## Diagnostic findings, Part 2

**Fig. 1:**
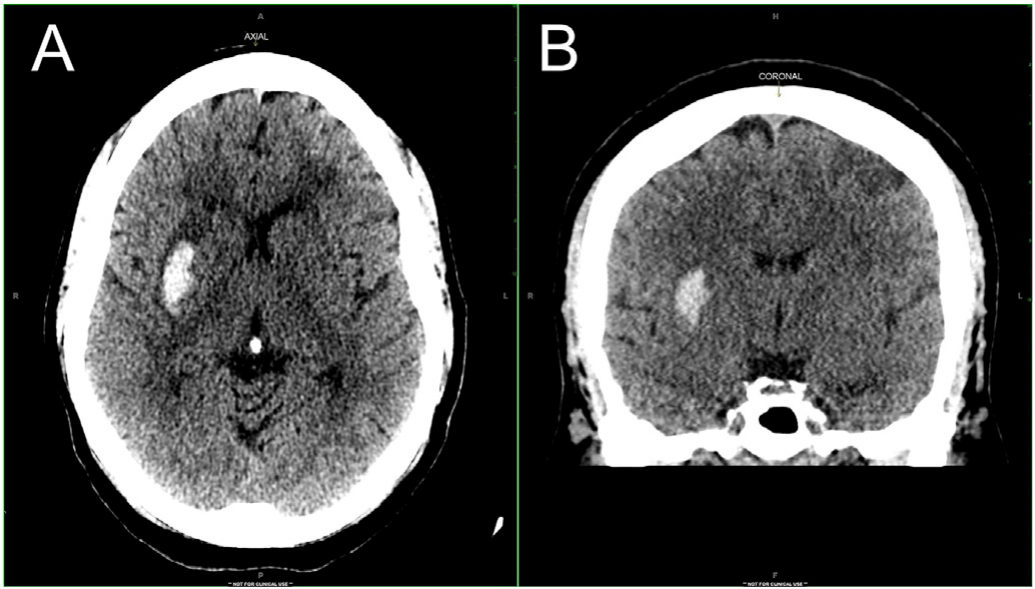
CT scan showing a right basal ganglion hemorrhage. (A) is an axial CT image that shows an ovoid shaped hyperdensity in the right putamen with minimal surrounding hypodensity, consistent with acute bleed surrounded by peripheral edema. (B) is a coronal CT image that shows an ovoid shaped hyperdensity in the right putamen, similar to the axial image. The images are diagnostic of a right-sided basal ganglia hemorrhage. CT, computed tomography.

Blood chemistry and hematologic studies reveal a normal PT/APTT/INR, a platelet count on the low end of normal, mild leukocytosis, and a negative toxicology screen. The results of laboratory investigations are detailed in Table 2, Table 3, Table 4. Initial workup for stroke includes *trans*-axial noncontrast CT imaging of the brain, which shows a hyperdense region in the right putamen part of the basal ganglia with minimal peripheral edema. These findings are consistent with hemorrhage as detailed in Fig. 1 A and B. Based on the findings, a diagnosis of hemorrhagic stroke is made.

**Table 2:** Laboratory investigations (complete hemogram) for the patient.

Test	Patient	Reference
RBC	4.82	(3.9–5.03 x10^12^/L)
WBC	10,700	(3500–10500/μL)
Hemoglobin	13.4	(12–15.5 g/dL)
Hematocrit	38 %	(34–44 %)
MCV	92	(81.6–98.3 fL)
MCH	28	(27.4–33.6 pg/cell)
MCHC	33.7	(33–35 g/dL)
RDW	12.10 %	(11.9–15.5 %)
Platelet count	152,000	(150,000–450,000/μL)

MCV, Mean Corpuscular volume; MCH, Mean Corpuscular Hemoglobin; MCHC, Mean Corpuscular Hemoglobin Concentration; RDW, Red cell Distribution Width; RBC, red blood cell; WBC, white blood cell.

**Table 3:** The patient's laboratory investigations (Complete metabolic and coagulation profile).

Test	Patient	Reference
Glucose, Serum	78	70–110 mg/dL
Blood Urea Nitrogen (BUN), Serum	3.7	5–25 mg/dL
Creatinine, Serum	0.54	0.12–1.06 mg/dL
estimated Glomerular Filtration Rate (eGFR)	65	>59 ml/min
BUN/Creatinine Ratio	6.85	8–27
Sodium, Serum	146	135–145 mmol/L
Potassium, Serum	3.3	3.5–5.2 mmol/L
Chloride, Serum	104	95–105 mmol/L
Calcium, Serum	8.1	8.7–9.8 mg/dL
Protein, Total, Serum	3.1	6–8 G/dL
Albumin, Serum	1.8	3.7–5.5 G/dL
Globulin, Total	1.3	1.5–4.5 G/dL
A/G Ratio	1.4	1.1–2.5
Bilirubin, Total	1.1	0.2–1.0 mg/dL
Alkaline phosphatase, Serum	368	145–320 IU/L
Aspartate aminotransferase (AST)	117	0–40 IU/L
Prothrombin Time (PT)	11.2	10.8–13.99 seconds
International Normalized Ratio (INR)	1	0.9–1.15
activated Partial Thromboplastin Time (aPTT)	28.1	21.1–33.89 seconds

**Table 4:** Toxicology screen for the patient.

Test	Patient result
Cannabinoid screen, urine	Negative
Phencyclidine screen, urine	Negative
Cocaine screen, urine	Negative
Methamphetamine screen, urine	Negative
Opiate, urine	Negative
Amphetamine screen, urine	Negative
Benzodiazepines, urine	Negative
Tricyclic antidepressant (TCA), urine	Negative
Methadone screen, urine	Negative
Barbiturate screen, urine	Negative
Oxycodone screen, urine	Negative
Propoxyphene, urine	Negative
Buprenorphine, urine	Negative

## Questions/discussion points, Part 2

### What are the most common causes of acute stroke?

Causes of stroke can be grouped into two categories: ischemic and hemorrhagic. Aging and hypertension are the two most important risk factors for stroke in general.^12^ Table 1 has a summary of the types of strokes and related pathologies. Ischemic stroke is much more common and accounts for about 85% of all cases. The most common cause of an ischemic stroke is thromboembolic disease or occasionally athero-embolic disease. Possible sources of emboli are the left atrium in the setting of atrial fibrillation, mitral valve vegetations, left ventricle thrombus due to stasis secondary to myocardial infarction, aortic valve vegetations, and the ascending aorta and carotid arteries in a patient with atherosclerosis. Other rare sources may include distant emboli, such as deep vein thrombosis in the setting of a patent foramen ovale known as a paradoxical embolus.^4^ Other comparatively rare causes include vasculitis, endocarditis, and cerebral venous disease.^2^ It is important to note that chronic hypertension can lead to segmental disorganization in the lenticulostriate arteries or fibrinoid necrosis weakening the arteriole causing edematous fluid to leak and damage the adjacent tissue. Thrombo-embolic occlusion of the artery is seen in addition to the surrounding tissue infarction. It mainly affects the deep arteries supplying the basal ganglia, resulting in a type of ischemic stroke known as a lacunar infarct.^13^, ^14^, ^15^ The so-called lacunar infarcts may be a result of incomplete infarction, old small hemorrhages, or as is sometimes argued, may be thromboembolic in nature.^12^^,^^15^

Hemorrhagic strokes comprise up to 15% of all strokes and can result from intracerebral hemorrhage due to rupture of intraparenchymal small blood vessels or may occur in patients with subarachnoid hemorrhage. The causes of intracerebral hemorrhage usually differ based on the location of the hemorrhage. Hypertension is the cause of more than 50% of all intracerebral hemorrhagic strokes. Additional risk factors that may lead to similar hemorrhagic small vessel disease are age and high cholesterol.^2^^,^^4^ The common causes of subarachnoid hemorrhage include trauma, rupture of a saccular aneurysm, vascular malformations, and several hematologic disturbances..^2^^,^^4^ Bleeding disorders or bleeding caused by extrinsic factors such as antithrombolytic therapy or anticoagulant therapy with attendant impaired blood clotting could also predisposes to and cause hemorrhagic stroke.^2^^,^^7^^,^^13^

### What are the most common locations of hypertensive hemorrhage?

**Fig. 2:**
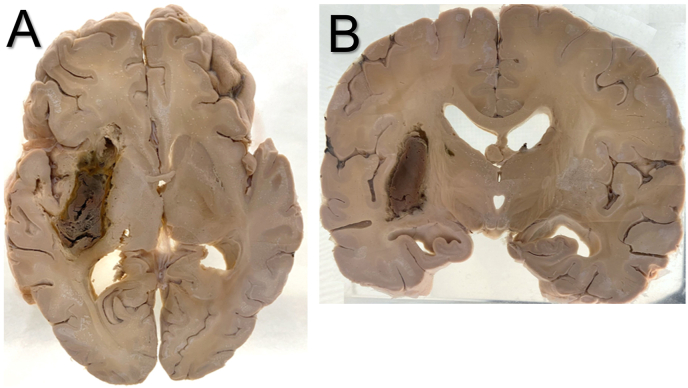
Gross pathology images of formalin-fixed, archival specimens demonstrating basal ganglia hemorrhages. Both axial (A) and sagittal (B) sections show hemorrhages in the right basal ganglion region closely resembling the radiology findings of the patient. (Image Courtesy Dr. Philip J Boyer, East Carolina University, Brody School of Medicine, Greenville, NC).

Basal ganglia and thalamic hemorrhages are characteristic locations of hypertensive hemorrhage and are thought to occur in the context of hypertension-related Charcot–Bouchard microaneurysms. Hemorrhagic stroke can be deep or lobar (Table 1). Lobar hemorrhages occur in the cerebral or cerebellar hemispheres. Deep hemorrhages occur in the basal ganglia, thalamus, and pons. Hemorrhages in the basal ganglia, especially the putamen, account for roughly 50–60% of cases of hypertensive intracerebral hemorrhage. Fig. 2 demonstrates the gross pathology that may be seen on autopsy. Other locations are less common and, in order of frequency, include the thalamus, pons, and cerebellar hemispheres (rarely).^13^ Around 2% of intracerebral hemorrhages involve multiple locations, which may be a sign of angiopathy or a bleeding disorder.^7^ Intraparenchymal hemorrhage confined to the occipital lobe is referred to as a lobar hemorrhage and is frequently caused by CAA.

### Which part of the cerebral vasculature is most affected by hypertension? Discuss pathophysiology and histopathology changes

**Fig. 3:**
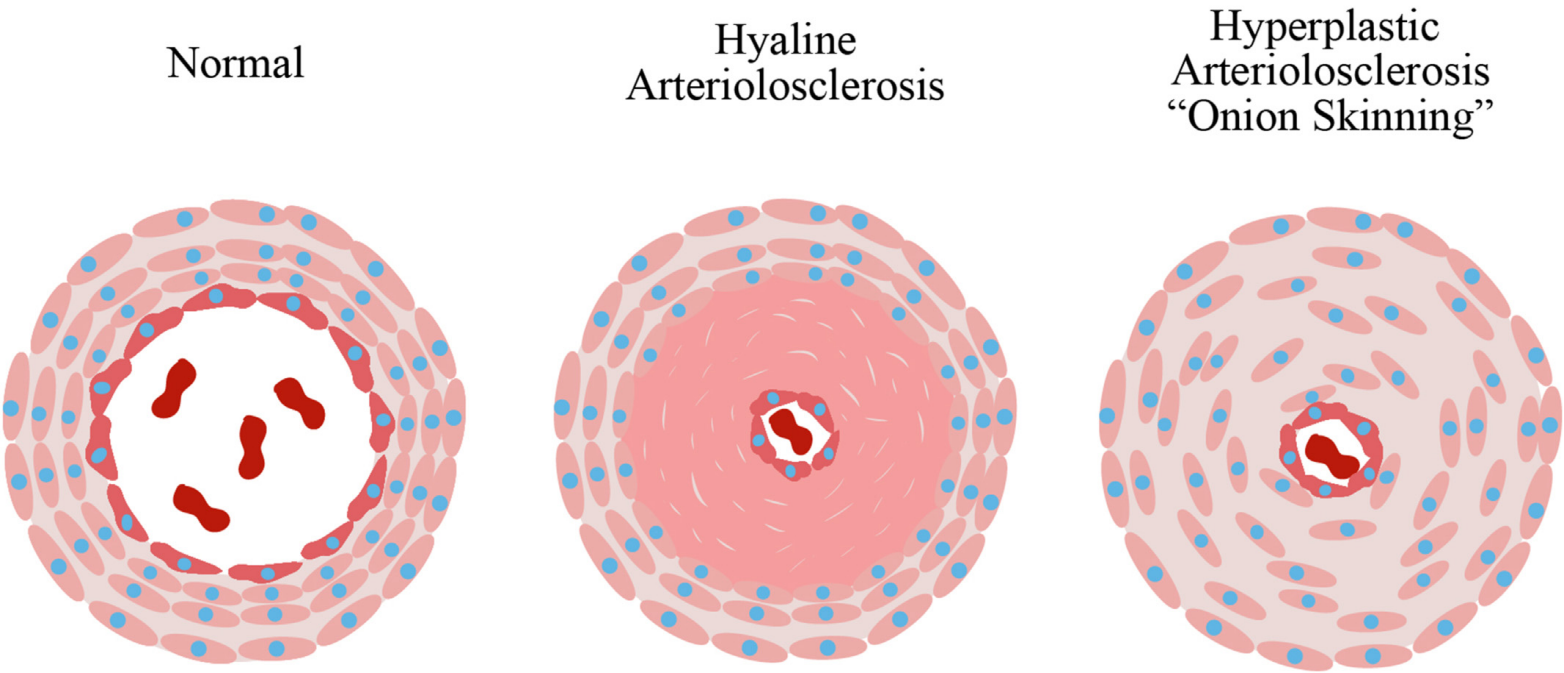
Effects of hypertension on blood vessels. The left panel shows a normal artery. The middle panel details hyaline arteriosclerosis. It is represented by the pink hyaline thickening of the media of the blood vessel wall, resulting in narrowing of the lumen. The panel on the right illustrates changes of hyperplastic arteriosclerosis. This is almost exclusively seen with severe hypertension causing concentric, thickening of the media of the vessel walls with narrowing of the lumen. It is most commonly seen in the renal vasculature.

The vessel wall changes that occur in chronic hypertension are most prominent in the small arterioles of the brain, especially the lenticulostriate arteries that supply the basal ganglia, hemispheric white matter, and brain stem.^2^^,^^5^^,^^13^ Autopsy studies of hypertensive brains suggest two types of lesions are responsible for stroke. Hypertension causes atherosclerosis in large arteries all over the body, including the cerebral arteries. It has been reported that hypertension tends to cause the development of atherosclerotic lesions into the smaller, more distal parts of the cerebral blood vessels.^12^ In these arteries (200–800 μm), the atheromatous plaques are either microatheroma (proximal perforating arteries), junctional atheroma (at their origin), or mural atheroma (circle of Willis). The mechanism of ischemia is mainly occlusive thrombosis or poststenotic hypoperfusion.^2^^,^^12^^,^^13^ The atheroma resembles common large vessel disease,^13^ but occlusive foam cell accumulation is also seen.^12^ The second type of lesion is hyaline arteriolosclerosis, which is a progressively destructive lesion seen in smaller vessels (40–300 μm).^12^ Arteriolosclerosis is associated with lacunar infarcts when the vessels are occluded and hypertensive intraparenchymal hemorrhage when the vessels rupture.^13^ Chronic hypertension can cause proliferative changes and necrosis of the arteriolar walls.^2^^,^^5^^,^^13^ The pathology consists of initial “onion skinning” of small vessels due to smooth muscle cell proliferation. Chronic high blood pressure in the vessel lumen forces plasma proteins into the arteriolar wall, causing hyaline arteriolosclerosis i.e., degeneration of smooth muscle cells causes the characteristic concentric hyaline wall thickening, loss of smooth muscle cells, and narrowing of the lumen of the small arteries and arterioles^12^^,^^13^ (Fig. 3). The weakened vessels are more prone to rupture causing hemorrhagic infarct.

**Fig. 4:**
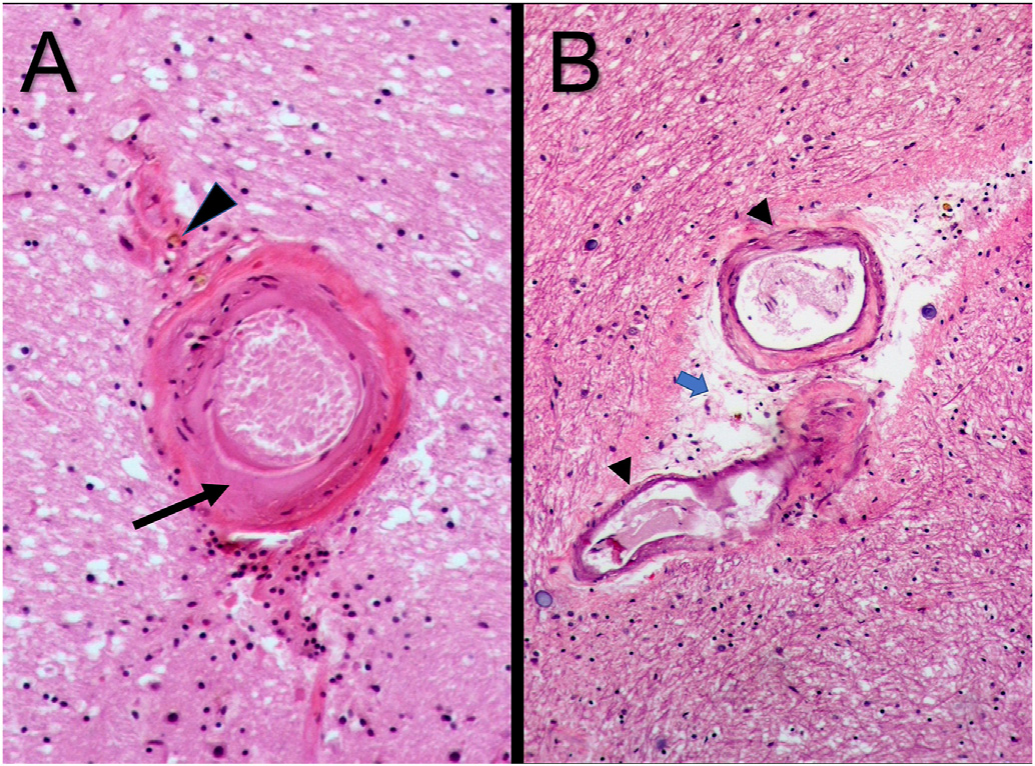
Histopathology of chronic hypertension resulting in cerebral hyaline arteriolosclerosis. (A) The photomicrograph shows microangiopathy in an arteriole. There is hyaline degeneration (arrow) along with edema of the surrounding areas. Hemosiderin-laden macrophages are frequently seen in the perivascular space around hypertensive vessels (arrowhead) (high power). (B) The photomicrograph shows two small vessels with fibrotic vessel wall (arrowhead) and expanded periarteriolar space due to edema (blue arrow) (high power magnification). These images are reproduced from Wikimedia Commons under the CC BY-SA 3.0 license: Fig. 4A at https://commons.wikimedia.org/wiki/File:Cerebral_Arteriolosclerosis,_HE.JPG and Fig. 4B at https://commons.wikimedia.org/wiki/File:Cerebral_Arteriolosclerosis,_HE_2.jpg.

Fibrinoid necrosis of the vessel wall, considered a hallmark of acute hypertensive cerebral vessel damage, is seen especially in malignant or accelerated hypertension. The presence of fibrinoid necrosis in hypertensive cerebral blood vessels suggests a selective vulnerability. This may be due to breach of the blood-brain barrier, edema, and leakage of plasma proteins into the vessel wall along with destruction of smooth muscle cells seen in arteriolosclerosis (Fig. 4A and B). This ultimately leads to blood pressure-induced breach of wall^3^^,^^7^ and cerebral hemorrhage.

### What is hyaline arteriosclerosis and how does it increase the risk of hemorrhage?

Hyaline arteriosclerosis is a common change in vessels of older patients with or without hypertension. However, chronic hypertension and diabetes mellitus result in generalized and severe changes. Damage to endothelial cells combined with chronic hypertension results in leakage of plasma proteins and increased smooth muscle cell matrix synthesis. Hyaline arteriosclerosis is most common in the vessels of the brain, eye, kidney, and peripheral nerves. Thus, hyaline arteriosclerosis leads to fibrosis and weakening of arteriolar walls, which affects the integrity of the wall with a resulting increase in vulnerability to rupture.^13^ Hyaline arteriosclerosis wall thickening and atrophy of the outer muscular layer results in loss of integrity of the wall, leading to the formation of false aneurysms called Charcot–Bouchard microaneurysms.^16^ Rupture of these microaneurysms is thought to be the major mechanism of hypertensive intracerebral hemorrhage.^7^^,^^13^^,^^16^

### Why does intracerebral hemorrhage cause neurologic deficits?

The escape of blood from vessels into cerebral tissue forms a growing mass that disrupts normal structure and anatomy. As bleeding continues and the mass grows, brain tissue adjacent to the bleed becomes distorted and compressed. Large hemorrhages can produce midline shift, putting the reticular activating system and respiratory centers at risk in the brainstem. Medium- and large-sized clots may interrupt the capsule of the putamen, whose key role is to regulate movement, causing hemiplegia of the contralateral side. Thalamic bleeds result in hemiplegia or hemiparesis due to damage to the adjacent internal capsule, the home of both motor and sensory fibers, causing contralateral sensory loss as a key clinical feature of thalamic hemorrhage. Ocular disturbances may occur due to the extension of a thalamic hemorrhage into the subthalamus or high midbrain, where ocular cranial nerves reside. Damage of these structures manifests as asymmetric gazes, vertical and lateral gaze palsies, forced downward deviation of the eyes, and absence of reaction to light. Pontine hemorrhage produces coma within minutes, causing total paralysis, rigidity, and small reactive pupils. Lateral eye movements elicited by turning or caloric response are absent, with death common within a few hours.^7^

### Discuss the changes that occur in a hematoma in the days to months following an intracerebral hemorrhage

The blood clot from an intracerebral hemorrhage undergoes a series of characteristic changes, beginning with clot formation within hours. About 24 hours after the hemorrhage, macrophages begin to phagocytose the extravasated red blood cells starting at the periphery of the hematoma. The clot gradually changes color from dark red to brown as the breakdown products of hemoglobin are produced and accumulate. The edema around the clot gets absorbed over the next days to weeks, and if large enough, may leave a cavity or scar in its place. In patients that survive, restoration of most motor function can occur after the hematoma is absorbed since original deficits are mostly due to mass effect rather than tissue damage. However, intracerebral hemorrhages generally have an unfavorable prognosis. If the original bleed was large or entered the ventricular system, the mortality rate is around 30% in the first 30 days due to a massive increase in intracerebral pressure compressing vital brain tissue.^7^

### How does the treatment for hemorrhagic stroke differ from ischemic stroke?

Treatment of acute ischemic stroke includes intravenous thrombolytic therapy if treatment is started within 4.5 hours of symptom onset. Mechanical thrombectomy is indicated for ischemic stroke within 24 hours of symptom onset if the stroke is due to a large artery occlusion, regardless of whether thrombolytic therapy was given. These treatments would worsen the extent of a hemorrhagic stroke and could potentially cause rebleeding, which is why it is important to distinguish ischemic versus hemorrhagic stroke with a CT scan prior to initiating treatment. Patients with ischemic stroke are also started on long-term antithrombotic therapy with aspirin and heparin/warfarin, a high-intensity statin medication, management of hypertension, and lifestyle modifications.^17^^,^^18^

Treatment of intracerebral hemorrhagic includes discontinuing any anticoagulant medications and use of reverse anticoagulation agents specific to each anticoagulant. These include administering coagulation factors and vitamin K in a patient who is taking warfarin or protamine sulfate in a patient who is on heparin. Blood pressure management is also important in patients with hemorrhagic stroke to prevent hemorrhage expansion. Blood pressure can become even more elevated in patients with baseline hypertension in the setting of hemorrhagic stroke secondary to elevations in intracerebral pressure. Systolic blood pressure goal with acute intracerebral hemorrhage includes rapid correction to under 220 mmHg, and gradual reduction to a target range of 140–160 mmHg. Management of intracranial pressure is also important in patients with acute intracerebral hemorrhage due to the risk of progressive brain compression and neurologic impairment. Elevated intracranial pressure in the setting of intracerebral hemorrhage can be due to mass effect of the initial hematoma or from its expansion after a rebleed, cerebral edema around the hematoma, or hydrocephalus from ventricular outflow obstruction. Patients with rapidly progressing impairment from increasing intracranial pressure may require surgical decompression. In patients who do not require surgical decompression, methods to reduce impairment from elevated intracranial pressure include elevating the head to 30°, mild sedation, antipyretics, and IV fluid replacement. Patients with intracerebral hemorrhage are also at increased risk of seizures posthemorrhage and should be managed with antiseizure medications accordingly. However, prophylactic antiseizure medication is not recommended in all patients.^17^^,^^18^

### Discuss the prognosis of patients with intracerebral hemorrhagic stroke

The 30-day mortality rate from intracerebral hemorrhage can range from 32–52% with half of the deaths occurring in the first two days after stroke. Risk factors for early mortality include advanced age, more severe initial clinical presentation, early use of “Do Not Resuscitate” orders and preceding use of antithrombotic agents. The clinical prediction score called the “ICH score” can be used to estimate 30-day mortality and includes the Glasgow coma scale score at presentation, initial hemorrhage volume, presence of intraventricular extension of the hemorrhage, presence of infratentorial hemorrhage, and age over 80 years old.^19^

Long-term prognosis focuses on functional recovery, cognitive impairment, and long-term mortality. Functional recovery has been found to be the most rapid in the first weeks to months after stroke with early rehabilitation maximizing recovery. The extent of functional recovery may be predicted using the ICH score discussed above. Cognitive impairment persists in 18–88% of patients with intracerebral hemorrhage. Patients with intracerebral hemorrhage have a decreased 10-year life-expectancy when compared to age- and sex-matched controls. The risk of recurrence of intracerebral hemorrhage is highest in the first 12 months after the initial stroke at about 1–9%. The risk of recurrence can be reduced with blood pressure management with a goal to maintain a blood pressure of less than 130/80 mmHg.^17^, ^18^, ^19^

### Discuss the cause of death and death certification in cases of stroke

This patient's cause of death can be attributed to complications of a right basal ganglia hemorrhage. This description uses a lumping strategy to include all of the pathologic processes, such as cerebral edema and swelling and others, involved with basal ganglia hemorrhage leading to death. The certification should also include other significant conditions contributing to death, which would include hypertension in this case. Another option for describing this patient's cause of death would be to list both cerebral edema/swelling and right basal ganglia hemorrhage as the cause. Hypertension would also be included as a significant condition contributing to death.

## Teaching points

• Intracerebral hemorrhage is the third most common cause of stroke after cerebral embolism and thrombotic disease and accounts for 15–20% of all stroke patients.
• Sudden onset of clinical features such as headache, vomiting, speech and language deficits (e.g. aphasia), hemiplegia or hemiparesis, and loss of consciousness are common signs of cerebral hemorrhage, helping distinguish it from ischemic stroke.
• Hypertension is one of the most common causes of intracranial hemorrhage, along with ruptured saccular aneurysms, vascular malformations, neoplasms (metastatic, and in some cases, primary), and anticoagulant use.
• Most hypertensive intracerebral hemorrhages occur in the putamen/basal ganglia and adjacent internal capsule. Other common locations are the thalamus, pons, and cerebellar hemispheres.
• Chronic uncontrolled hypertension leads to the development of hyaline arteriosclerosis, fibrinoid necrosis, and Charcot–Bouchard microaneurysms in small arterial walls, most commonly in the lenticulostriate arteries of the basal ganglia.
• Formation of Charcot–Bouchard microaneurysms is also associated with chronic hypertension and can occur in vessels that are less than 300 μm in diameter, including those in the basal ganglia.
• Extravasation of blood into the surrounding brain tissue typically results from rupture of Charcot–Bouchard aneurysms or fibrinoid necrosis of the vessel wall, leading to compression of nearby tissue by the hematoma and subsequent edema. Brainstem herniation or brainstem compression, coma, and death can occur.
• If the patient survives the acute episode, over the next few weeks the clot is broken down and phagocytosed by histiocytes. Methemoglobin can be seen around the periphery of the hematoma.
• Two to three months after a bleed, the hematoma is slowly absorbed, leaving a cavity surrounded by glial scar with residual hemosiderin-laden macrophages in its place.

## Funding

The author(s) received no extramural financial support for the research, authorship, and/or publication of this article.

## Declaration of competing interest

The authors declare no potential conflicts of interest with respect to the research, authorship, and/or publication.

There are no financial disclosures and no funding or financial incentives were received for support, research, authorship and/or publication of this manuscript.

## References

[1] Knollmann-Ritschel B.E.C., Huppmann A.R., Borowitz M.J., Conran R. (2023). Pathology competencies in medical education and educational cases: update 2023. Acad Pathol.

[2] Langhorne P., Ralston S., Penman I., Strachan M., Hobson R. (2018). Davidson's Principles and Practice of Medicine.

[3] Marcolini E., Stretz C., DeWitt K.M. (2019). Intracranial hemorrhage and intracranial hypertension. Emerg Med Clin.

[4] Smith W., Hemphill I.J., Johnston S., Loscalzo J., Fauci A., Kasper D., Hauser S., Longo D., Jameson J. (2022). Harrison's Principles of Internal Medicine.

[5] Grinberg L.T., Thal D.R. (2010). Vascular pathology in the aged human brain. Acta Neuropathol.

[6] Montaño A., Hanley D.F., Hemphill J.C. (2021). 3rd. Hemorrhagic stroke. Handb Clin Neurol.

[7] Ropper A.H., Samuels M.A., Klein J.P., Prasad S., Ropper A.H., Samuels M.A., Klein J.P., Prasad S. (2019). Adams and Victor's Principles of Neurology.

[8] Caplan L, Kasner S, Dashe J. Overview of the evaluation of stroke. *UpToDate.* Publishers Walters Kluwer*.* Accessed April 5, 2022. https://www.uptodate.com/contents/overview-of-the-evaluation-of-stroke.

[9] Kakkar P., Kakkar T., Patankar T., Saha S. (2021). Current approaches and advances in the imaging of stroke. Dis Model Mech.

[10] Sanders B.D., Davis M.G., Holley S.L., Phillippi J.C. (2018). Pregnancy-associated stroke. J Midwifery Wom Health.

[11] Kernan W.N., Viscoli C.M., Brass L.M. (2000). Phenylpropanolamine and the risk of hemorrhagic stroke. N Engl J Med.

[12] Alistair G. (2002). Hypertensive cerebral small vessel disease and stroke. Brain Pathol.

[13] Margeta M., Perry A., Kumar V., Abbas A., Aster J., Robbins S. (2021). Robbins & Cotran Pathologic Basis of Disease.

[14] Tapia J.F., Kase C.S., Sawyer R.H., Mohr J.P. (1983). Hypertensive putaminal hemorrhage presenting as pure motor hemiparesis. Stroke.

[15] Wardlaw J.M. (2005). What causes lacunar stroke?. J Neurol Neurosurg Psychiatr.

[16] Gupta K., J M.D. (2024). https://www.ncbi.nlm.nih.gov/books/NBK553028/.

[17] Powers W.J., Rabinstein A.A., Ackerson T. (2018). 2018 guidelines for the early management of patients with acute ischemic stroke: a guideline for healthcare professionals from the American heart association/American stroke association. Stroke.

[18] Greenberg S.M., Ziai W.C., Cordonnier C. (2022). Guideline for the management of patients with spontaneous intracerebral hemorrhage: a guideline from the American heart association/American stroke association. Stroke.

[19] Rordorf G., McDonald C. (2001). https://www.uptodate.com/contents/spontaneous-intracerebral-hemorrhage-acute-treatment-and-prognosis?topicRef=1133&source=see_link.

